# Emergency Medicine Resident Needs Assessment and Preferences for a High-value Care Curriculum

**DOI:** 10.5811/westjem.59622

**Published:** 2023-12-08

**Authors:** Bennett H. Lane, Simanjit K. Mand, Stewart Wright, Sally Santen, Brittany Punches

**Affiliations:** * University of Cincinnati College of Medicine, Department of Emergency Medicine, Cincinnati, Ohio; † University of Cincinnati Health Air Care & Mobile Care, Cincinnati, Ohio; ‡ Ohio State University, Colleges of Nursing and Medicine, Department of Emergency Medicine, Columbus, Ohio; ° Bennett H. Lane and Simanjit K. Mand contributed equally to this work

## Abstract

**Introduction:**

Consideration of the cost of care and value in healthcare is now a recognized element of physician training. Despite the urgency to educate trainees in high-value care (HVC), educational curricula and evaluation of these training paths remain limited, especially with respect to emergency medicine (EM) residents. We aimed to complete a needs assessment and evaluate curricular preferences for instruction on HVC among EM residents.

**Methods:**

This was a qualitative, exploratory study using content analysis of two focus groups including a total of eight EM residents from a single Midwestern EM residency training program. Participants also completed a survey questionnaire.

**Results:**

There were two themes. Within the overall theme of resident experience with and perception of HVC, we found five sub-themes: 1) understanding of HVC focuses on diagnosis and decision-making; 2) concern about patient costs, including the effects on patients’ lives and their ability to engage with recommended outpatient care; 3) conflict between internal beliefs and external expectations, including patients’ perceptions of value; 4) approach to HVC changes with increasing clinical experience; and 5) slow-moving, political discussion around HVC. Within the overall theme of desired education and curricular design, we identified four sub-themes: 1) limited prior education on HVC and health economics; 2) motivation to receive training on HVC and health economics; 3) desire for discussion-based format for HVC curriculum; and 4) curriculum targeted to level of training. Respondents indicated greatest acceptability of interactive, discussion-based formats.

**Discussion:**

We conducted a targeted needs assessment for HVC among EM residents. We identified broad interest in the topic and limited self-reported baseline knowledge. Curricular content may benefit from incorporating resident concerns about patient costs and conflict between external expectations and internal beliefs about HVC. Curricular design may benefit from a focus on interactive, discussion-based modalities and tailoring to the learner’s level of training.

Population Health Research Capsule What do we already know about this issue?

*Resident education guidelines now incorporate the topic of value in health care, but few resident-focused needs assessments for this concept are available.*

What was the research question?

*For high-value care, what are emergency medicine residents’ needs, interests, and preferences for instructional modality?*

What was the major finding of the study?

*Residents self-report low knowledge but are interested in education on high-value care. They prefer discussion-based modalities.*

How does this improve population health?

*Addressing cost of care through graduate medical education may help address accessibility and affordability of care.*

## INTRODUCTION

A recent shift to focus on “value” in healthcare, often defined as health outcomes achieved per dollar spent, has emerged in response to persistently rising costs over decades.^1^ Recent events have highlighted the cost of emergency care in the national spotlight, including federal legislation on surprise billing, insurer denials of claims for emergency department (ED) visits without a final emergent diagnosis, and regulations on payments for air ambulance transports.^2^ ^–^ ^5^ Consistent with these developments, current Accreditation Council for Graduate Medical Education (ACGME) guidelines state that “residents must demonstrate competence in … incorporating considerations of value, equity, cost awareness, delivery and payment, and risk-benefit analysis in patient and/or population-based care as appropriate.”^6^

Despite the current ACGME guidelines and increasing demands for high-value care (HVC), the appropriate educational content and instructional methods have not been clearly established. Moriates and colleagues delineated 21 HVC competencies with beginning, proficient, and expert levels through an iterative process led by a multidisciplinary committee.^7^ While rigorous and expert-led, this approach did not include a resident-focused needs assessment, and subsequent needs evaluations have been limited to surveys of internal medicine or pediatrics residents at a single site.^8^ ^–^ ^10^ Similarly, evaluation of proposed internal medicine or pediatrics resident curricula have been limited to single-site pre-/post-surveys, with one study also including post-implementation focus groups.^10^ ^–^ ^13^

Within emergency medicine (EM), HVC and health economics educational resources are limited, as a 2010 systematic review of cost-effectiveness curricula identified only a single EM curriculum focused on the Ottawa ankle rules. Since that review, two additional contributions that we are aware of include 1) the Emergency Medicine Residents’ Association *Residents’ Advocacy Handbook* addressing policy-related topics in a textbook-like format and 2) a cost-conscious care curriculum developed by Lin and Laskowski at a single site in New York (personal communication, L. Laskowski).^14^ ^,^ ^15^ There is a paucity of formal, resident-focused needs assessments across specialties, particularly in EM. Our objective was to perform a targeted needs assessment to assess EM residents’ needs and interests in HVC and preferences for instructional modality.

## METHODS

### Study Design

As part of a curriculum development process, we performed a problem identification and targeted needs assessment for EM residents, corresponding to Kern’s six-step approach to curricular development.^16^ To achieve our objective, we conducted a qualitative, exploratory study using conventional content analysis. This method allowed us to critically examine the participant responses to identify common categories and elucidate themes. Our secondary objective to determine preferences for instructional modality included a collection of respondents’ self-assessments using a survey questionnaire. We obtained institutional review board (IRB) approval for all study procedures.

### Setting and Participant Selection

The setting was a single Midwestern United States EM residency program with 56 total residents. Two physician authors were residents at the time of the data collection phase of the project (BHL, SKM). Recruitment of a convenience sample of eight EM residents was performed via email by one of the authors (SKM) to the remaining 54 residents. Nine residents responded. (One resident could not participate due to scheduling constraints.) No participant terminated their participation during the focus group.

### Data Collection Procedures

We obtained documentation of informed consent prior to study procedures. A semi-structured interview guide for focus groups was primarily authored by a single author (BHL) and reviewed sequentially by additional authors for revision of content and phrasing (SKM, BP). The interview guide is included as Appendix 1. Focus groups were co-led by two physician authors who were residents at the time (BHL, SKM) following the interview guide. Both focus groups were audio recorded and subsequently transcribed. No field notes were made, nor were transcripts returned to participants for comment. The focus groups occurred during September 2020 in a medical school conference room with no other person present aside from focus group leaders and participants. After the focus group discussion was complete, participants independently completed a survey questionnaire using Likert scale and rank order questions on paper (Appendix 2). Each focus group included four participants with at least one intern (postgraduate year [PGY] 1) in each group. In total, the first group included one PGY-1, one PGY-2, and two PGY-3 residents; the second focus group included two PGY-1 and two PGY-4 residents. Each focus group lasted between 75–85 minutes. No repeat interviews were completed. Participants received a $15 gift card for compensation, consistent with IRB guidelines.

### Data Analysis

The transcripts were reviewed and conventional content analysis with line-by-line coding was completed by two independent coders (BHL, SKM). Using an open coding technique, important statements were identified (generally termed “the first cut”).^17^ Codes were developed in vivo and did not reference previous literature. (They are depicted in a coding tree in Appendix 3.) Significant redundancy in codes was identified, which was felt to be consistent with thematic saturation.^18^ The analysis team came together with a third reviewer (BP) to categorize, refine, and cluster important statements, and subsequent themes and domains emerged. We used the consolidated criteria for reporting qualitative research (COREQ) as reporting guidelines (Appendix 5).^19^ Descriptive statistics were performed in Microsoft Excel for questionnaire data, and we used Word (Microsoft, Redmond, WA) for transcripts and coding documentation. The use of independent coders and a team of three to categorize and develop themes enhanced credibility, and investigator triangulation aided confirmability of the results.^18^

### Reflexivity Statement

Reflexivity of the research team included recognition that the focus group leaders and coders were known to the participants and identified their respective specific interests in HVC/health economics (BHL) and medical education (SKM) to the participants as part of the introduction. The focus group leaders identified as male (BHL) and female (SKM). BHL and SKM were residents at the time of the study. BP provided training to BHL and SKM regarding techniques in semi-structured, focus group facilitation; BHL had limited prior experience with focus group facilitation. A methodological limitation is that the same residents comprised the focus groups and completed survey questionnaires; survey questionnaire results may have been influenced by the preceding focus group discussion, although all questionnaires were completed independently by all participants without additional discussion.

## RESULTS

A total of eight residents participated. With respect to the importance of education about HVC topics, residents endorsed the relevance of HVC topics to the resident physician (7/8, [88%]) and the importance of a HVC curriculum (8/8, [100%]) (Appendix 4, Figure 2). We identified two overarching themes: 1) experience with and perception of HVC; and 2) desired education and curricular design. For each overarching theme, component sub-themes summarized clusters of resident comments for which we include representative comments and (if identified) participant recommendations.

### Overarching Theme 1: Experience with and Perception of High-value Care

#### Sub-theme 1: Understanding of high-value care focuses on diagnosis and decision-making.

Residents most frequently associated HVC with the activities that facilitate diagnosis and decision-making in the ED. For example, when asked whether they had a general definition for or had heard of the phrase “high-value care,” one resident highlighted using the ED evaluation to “*appropriately figure out what is going on with this patient and decide where to send them”* (Resident #1, PGY-1). In this understanding, residents believe care activities are high value if they allow the clinician to make a diagnosis or disposition. Less commonly, other residents mentioned aspects of HVC such as resource use, stewardship (citing a specific example of a cost-savings initiative related to the use of combat gauze [Resident #7, PGY-4]), and the concept of cost-benefit analysis: “*clinical decision rules that … reduce unnecessary head CTs, not only from a radiation perspective, but also from a cost-savings perspective”* (Resident #8, PGY-4).

#### Sub-theme 2: Concern about patient costs.

In the focus group discussion, residents voiced uncertainty due to varying patient insurance reimbursement of care provided in the ED and concerns surrounding high patient costs, in large part due to a self-identified lack of knowledge. Because of this knowledge gap, residents felt inadequately prepared to have conversations surrounding cost and insurance coverage with patients. One of the participants recalled a patient encounter in which the resident felt uninformed to address the patient’s reaction after the resident disclosed the presence of a new mass concerning for cancer:

*How much is this going to cost me? How am I going to pay for this?’[and] I didn’t know the answer. … It’d be nice if I actually had some data … like you’re uninsured, it’s ok, because it’s going to be like this for the financial plan, if you’re insured, this is what happens. I have no clue.”(Resident #5, PGY-3)*

Other residents stated that they were unaware of the costs of commonly ordered diagnostics and therapeutics in the ED. They described being concerned and unaware of the financial and social ramifications of care activities on patients’ lives outside of the hospital, and they particularly worried about the impact on patients’ ability to engage with recommended outpatient care: “*It’s how much the patient gets charged that would actually matter from a social determinants of health perspective*” (Resident #2, PGY-1). Residents particularly cited feeling challenged by shared decision-making discussions when patients had financial concerns.

#### Sub-theme 3: Conflict between internal beliefs and external expectations.

Residents noted that there may be a conflict between a physician’s personal beliefs and the external expectations and pressures they face. Some external expectations, such as those from systems-level “hurdles” placed in the electronic health record-ordering interface, are explicitly identifiable for residents: “*I try to order [intravenous acetaminophen] all the time. IT takes you through, you have to go through all these questions because they’re trying to keep me from ordering [it]. … I know they’re trying to keep me from ordering it, but I’m going to keep on ordering it*” (Resident #8, PGY-4). Other external expectations are perceived to be implicit within the medical community: *“Even though we talk about in an academic setting, or in a boardroom, it’s OK to have a miss from a statistical perspective, I think culturally that’s not acceptable. … It’s just not playing out in the real world, in my opinion, accepting that there is a miss rate*” (Resident #4, PGY-2).

Residents particularly highlighted that patients are a source of external expectations and recognized that patients may view cost, quality, and value of care differently from the emergency physician. This difference in perception may lead to a disconnect in expectations: *“Value can really be in the eyes of the beholder … makes me think about what I think might be the best thing for the patient may not be at all the same as what the patient values”* (Resident #6, PGY-3). Moreover, the conflict between internal beliefs and external expectations can overshadow attempts to prioritize HVC. A context cited for this conflict were ED visits of patients who commonly frequent the ED. For these patients, the lack of community resources for patients can be frustrating and render a learner feeling helpless or unable to provide holistic patient care. For these patients, trainees noted feeling a disconnect between the care they felt expected to provide and the care they desired to provide.

#### Sub theme 4: Approach to high-value care changes with increasing clinical experience.

Residents shared anecdotes that demonstrate how the definition of and approach to optimize HVC changes with increasing clinical experience. One junior resident highlighted “wanting to know” as motivation for ordering testing: *“I’m as curious as [the patients] are, to be honest; so I want to know that this patient is perhaps a presentation of [a specific diagnosis]” (Resident #4, PGY-2).* Similarly, as one non-intern resident reflected:

*“And honestly, that’s something that comes with time – like if you told me as an intern I could order a million-dollar test and get the answer that I need, I would 100% do it because it’s easy, I’ll be right, and I can help the patient. But as you practice medicine you realize … if you have a million-dollar test to answer if it’s GERD … it’s not going to change your management … As I’m progressing through residency I get more and more curious, and I’m more willing to accept information about [HVC]” (Resident #5, PGY-3)*

#### Sub-theme 5: Slow-moving, political discussion around high-value care in medicine.

In general, residents describe themselves as loosely aware of the political, academic, financial, and clinical implications of national discussions on HVC topics for future emergency physicians. For example, “*How you determine value? I remember back when Obama was still around and in office, I remember that was a big discussion, you know—what is real value and who determines that? That’s sort of a black box” (Resident #8, PGY-4).* Another resident reflected,

*“There is always chatter out there in the … political and insurance world. And I’m not sure I know where like the landmark policy or … guiding foundation is for that conversation. So, certainly, outside there is a feeling that there is always this chatter happening*” (Resident #4, PGY-2).

When asked about proposed physician reimbursement models currently undergoing federal regulatory review, most residents did not know what those future policies entailed. In addition, many residents reported not being well versed in current reimbursement models, although non-intern residents reported more interest in current reimbursement information.

### Overarching Theme 2: Desired Education and Curricular Design

#### Sub-theme 1: Limited medical education on health economics and high-value care.

When asked about their prior training in health economics and HVC topics, all residents noted minimal to no prior exposure during their medical training. In the survey questionnaire, all participants (8/8 [100%]) either strongly disagreed or disagreed with the statement “I feel confident that I know the cost of the care that I provide to patients in the emergency department” (Appendix 4). Much of the prior exposure described by residents was comprised of brief and infrequent didactic-based discussions that were described as leading to limited information retention and limited application to clinical practice.

Beyond this, they voiced the belief that there were few opportunities for knowledge acquisition due to lack of available resources, particularly with respect to prices and costs of healthcare activities. Residents were not familiar with hospital-based or nationally based resources that could assist with day-to-day clinical healthcare questions such as patient cost: “*I think hospitals are mandated to have some sort of list, master list, of how much things costs, but it’s also super hard to find … I have no idea where I would find that information*” (Resident #7, PGY-4).

#### Sub-theme 2: Motivated to receive training on high-value care and health economics.

The EM residents identified themselves as frontline healthcare workers. In their role, they interact directly with the community and patients with diverse backgrounds, particularly individuals facing financial barriers to accessing care. Because of this unique position in the medical field, residents believe that financial and insurance pressures may underlie patients’ utilization of the ED and that clinicians should therefore understand these factors. One resident reflected

“*I think when you … look … at healthcare as a gestalt, people are seeing primary care [clinicians] less and less and relying on the ED more for primary care. And assuming that that trend continues … I think as an emergency physician it is important to know those things [healthcare economics topics] because of that reason, just the utilization of the ED in general*” (Resident #1, PGY-1).

One resident also noted that the lack of health economics knowledge can put emergency physicians at a disadvantage in influencing and leading systems-based practice:

*“I think not understanding [HVC and health economics topics] takes away a lot of our power to be a leader and makes us more pawns carrying out someone else’s vision of how medicine should be practiced” (Resident #6, PGY-3).*

Residents recognized the importance of and need for further training on HVC topics to understand the impact that their decision-making has on patients and the healthcare system.

#### Sub-theme 3: Desire for discussion-based format for high-value care curriculum.

Residents were asked what the optimal format for HVC curriculum would be for residency-level learners, and the majority were in support of a discussion-based format.

*“I like the idea of the case-based, small-group discussion. Especially when you have attendings there, and you have varied learner levels, and I kind of like that because you get varied sorts of inputs and that’s interesting. And I just feel like this sort of stuff, these sorts of topics, are best, for me, explored verbally” (Resident #8, PGY-4).*

One resident noted that because this is not common knowledge among emergency clinicians, involving a content expert would be critical to a successful curriculum:

*“Another part of incorporating this, is who is the content expert…. [HVC care is] a topic that … a typical academic [emergency] physician would [not] know about. It almost needs to be a collaboration… [someone] with health economic interest and knowledge and someone with an education background, too, to figure out how to incorporate this” (Resident #7, PGY-4).*

**Figure 1.**
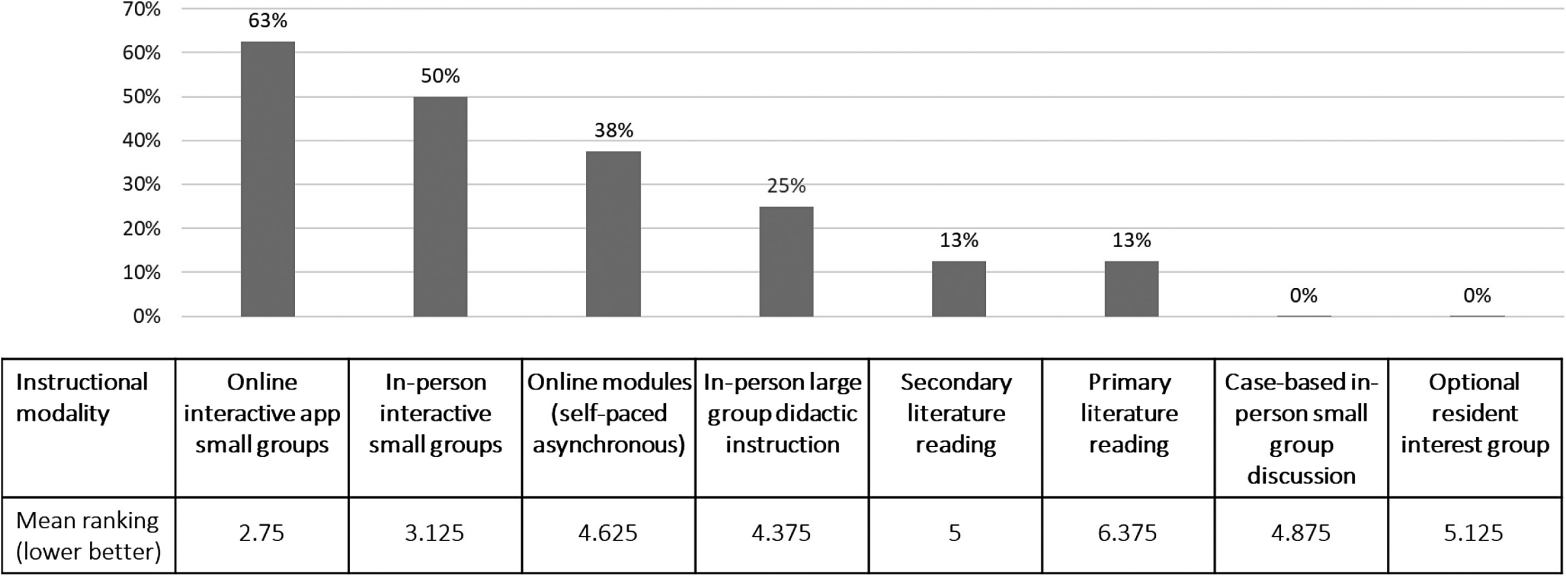
Percentage of respondents ranking each instructional modality among top two choices and mean ranking within eight modality options (*n* = 8).

Consistent with this qualitative theme, the highest percentage of residents ranked modalities with the opportunity for interactive small-group discussion highly, whether as online apps or in person, on the survey questionnaire (Figure 1).

#### Sub-theme 4: Curriculum targeted to their level of training.

While most of the residents recognized the need for a formal HVC curriculum during medical training, there was variation in when they thought this curriculum should be introduced at the residency-training level. The PGY-1 and PGY-2 residents voiced desire to focus on clinical knowledge acquisition in lieu of health economics topics:

*“As an intern, I’d rather be more towards the clinical aspect of things right now … I don’t think I’ve developed that skill enough to want to sacrifice one of those journal clubs for health economics. I think as a later resident, I’d be on board …” (Resident #1, PGY-1).*

*“My initial thought was that I would want something clinically relevant because I feel like [I am] earlier in training and just trying to build that foundation …” (Resident #4, PGY-2).*

A non-intern resident noted *“I feel like as I’m progressing through residency, I get more and more curious and I’m more willing to accept information about that stuff [HVC]”* (Resident #5, PGY-3). In reply to an intern indicating the topic of “[relative value units] *and physician-associated income … wouldn’t appeal or apply to me right now when I would just forget it*” (Resident #3, PGY1), Resident #7 (a PGY-4) reflected that non-intern residents would be interested due to personal decision-making: *“I would say the PGY-3s because some of the PGYs would start signing contracts in the summer”*

An interesting perspective raised by one of the non-intern residents was the potential to negatively influence junior learners’ practice patterns if topics of HVC were introduced too early in medical training:

*“I wonder from an education mission side, could you influence early trainees’… practice patterns because of knowledge of this. And I don’t want that to happen … you need to see where you fall in that spectrum to develop your practice pattern. And I wonder if you find out that a test costs this amount of money, maybe you won’t get to fully explore that spectrum and develop your own point on that spectrum” (Resident #7, PGY-4).*

Lastly, a couple of residents voiced concern about the integration of a novel curriculum in an EM training program given that EM’s scope of practice already addresses many adjacent disciplines:

*“We’re all kind of in agreement that a baseline level of understanding you should have … but as far as about data and literature … I’d kind of reserve that for people that have an interest in it, similar to how we do with other things, like sports medicine” (Resident #3, PGY-1).*

*“You have so many things to learn. Not only clinically, but also our non-clinical curriculum … is pretty impressive, so it’s tough [to] add a whole other curriculum” (Resident #8, PGY-4).*

## DISCUSSION

Residents recognized the importance of learning HVC principles for application in both patient care and to inform systems-based practice; however, they felt inadequately trained on the topic. Our needs assessment identified two main themes to inform EM-specific curricula addressing HVC topics: resident experience with and perception of HVC, and desired education and curricular design.

Consistent with studies in other disciplines and settings, the residents reported limited confidence in their knowledge of basic HVC principles, and the financial impacts of cost of care for individual patients and the healthcare system as a whole.^9^ ^,^ ^10^ ^,^ ^13^ Sub-themes 1 (understanding of HVC focuses on diagnosis and decision-making) and 2 (resident concerns about patient costs) in this study were consistent with themes from focus groups completed with general pediatrics residents at two centers of “how an intervention changes management” and “thinking about the cost as a harm.”^10^

Residents stated that early on in their training, HVC knowledge gaps are related to patient costs, patient insurance reimbursement, cost-benefit analysis, and resource stewardship. Later, self-identified knowledge gaps emerging as non-intern learners were primarily related to physician reimbursement. A review of the literature, including prior work within pediatrics and internal medicine, suggested no prior evidence of resident knowledge or interest varying by experience level; if validated in additional settings, such variations with learner experience would provide valuable guidance in the design of educational curricula.

The resident participants stated their lack of formal training in and basic knowledge of HVC was a barrier to providing high-value emergency care. They also reported limited awareness of national health policy yet were less interested in a detailed understanding of these topics. This finding suggests that a specialized elective may be better suited to education regarding health policy topics that do not directly tie into day-to-day emergency care, as in the example described by Greysen and colleagues.^21^ Finally, the participants also indicated the need for more education on system-wide reimbursement and HVC policies. To meet this need, prior national-level survey data from internal medicine residents and program directors suggests that institutional support for both HVC faculty development and provision of physician cost-of-care performance data are associated with an increase in resident reports of education on HVC.^22^

Unanticipated aspects of HVC that were viewed as learner obstacles included dynamic conflicts between internal learner beliefs and external expectations and the variability in value perception between patients and clinicians. These issues may complicate residents’ perception of and implementation of HVC in the clinical setting; addressing these issues within HVC education is critical to avoid unintentional creation of anxiety, or even moral distress, in the training environment. In an intern-targeted curriculum in internal medicine, Hom and colleagues also discussed resident-perceived barriers surrounding intra-team, interdisciplinary, and patient and family dynamics and how they complicate understanding and implementation of HVC principles at an early learner stage.^14^ Thus, future curricula will need to focus both on foundational knowledge dissemination and techniques on how to approach the above barriers.

An additional unexpected barrier raised by residents in the focus group was the concern that the existing EM training curriculum does not have the capacity to incorporate HVC; and, therefore, HVC training may not fit as a core element. While not addressed in these focus groups, a future direction for work in this area should include evaluation of how residents would weigh HVC training compared to other curricular elements and whether there would be opportunity to make potential “tradeoffs.”

In terms of curricular design and format, themes emerged to optimize not only knowledge acquisition and understanding, but also timing during the residency training program. The resident participants were in support of an expert-led, discussion-based curriculum to learn the principles of HVC, consistent with the experiences of Hom and colleagues.^13^ These findings also coincide with those of Stammen et al in their systematic review, concluding that reflective practice through feedback and group discussions incentivize physicians to think critically about medical decisions.^19^The residents also suggested that HVC topics should be targeted more toward non-intern residents who have mastered proficiency in basic clinical knowledge and skills and would be able to apply these new principles with more purpose than their junior counterparts, although some earlier knowledge base to supplement formative experiential growth throughout residency may be beneficial. They did voice concern that the introduction of HVC too early in residency could jeopardize early learners’ practice pattern development.

## LIMITATIONS

There are several limitations to consider with regard to our study. First, this study reflects a sample of residents from a single-center, large academic hospital and may not be applicable to all academic- and community-based training programs. Because it was a single-center study, we could not distinguish how three-year programs or four-year programs with different approaches to resident progression or “seniority” would differ from the findings identified here. Second, only a small subset of program residents participated in either focus group, leading to the possibility of selection bias with regard to the participants who volunteered to discuss their thoughts on HVC. These residents may have had a particular interest in medical education or HVC that may not be applicable to all EM residents across the country. The small subset of participating residents also likely limited the number of available perspectives to be collected and inform thematic saturation.

Third, the study included a mix of junior and senior residents. While the study allowed for a rich spectrum of experience to inform previous exposure to HVC principles, it may not have been as impactful as evaluating the perspectives of the most experienced residents in a program who had nearly completed the entire program curriculum and could identify areas for nuanced improvement. Fourth, while the use of focus groups (rather than one-on-one interviews) allowed emergent discussion between participants, the presence of peers may have led some participants to avoid making statements due to fear of being perceived as controversial. Fifth, due to transitions in roles, member checking could not be performed. While our study adds a critically necessary needs assessment to the current body of literature, further and more rigorous studies that include a larger number of residency programs and participating residents are needed to verify these findings to accurately inform future EM curricula.

## CONCLUSION

Our targeted needs assessment indicates that residents currently face gaps in knowledge of high-value care topics pertaining to the medical care that they provide and may benefit from additional training during residency. Residents interviewed in this study identified several perceived barriers to understanding HVC, but they consistently expressed interest in a formal curriculum to address these challenges. We found a preference for interactive, small-group discussion-based formats with content adjusted by level of clinical training.

## Supplementary Information





## References

[1] Porter ME . What is value in health care? N Engl J Med. 2010;363(26):2477–81. 21142528 10.1056/NEJMp1011024

[2] Kliff S Sanger-Katz M . For surprise medical bills, it’s the beginning of the end. *The New York Times*. Published July 1, 2021. Available at: https://www.nytimes.com/2021/07/01/upshot/surprise-medical-bills-biden.html. Accessed July 19, 2021.

[3] Chou SC Gondi S Baker O et al . Analysis of a commercial insurance policy to deny coverage for emergency department visits with nonemergent diagnoses. JAMA Netw Open. 2018;1(6):e183731. 30646254 10.1001/jamanetworkopen.2018.3731 PMC6324426

[4] Kelly M . Sky-high air ambulance prices. Ann Emerg Med. 2020;76(5):A17–A20. 34842164 10.1016/j.annemergmed.2020.09.447 PMC7575426

[5] Mole Beth . Biggest health insurer plans to deny ER bills if it doubts you had an emergency. *ArsTechnica*. Published June 10, 2021. Available at: https://arstechnica.com/science/2021/06/biggest-health-insurer-plans-to-deny-er-bills-if-it-doubts-you-had-an-emergency/. Accessed July 19, 2021.

[6] Accreditation Council for Graduate Medical Education . ACGME Common Program Requirements (Residency). Published online July 1, 2023. Available at: https://www.acgme.org/globalassets/pfassets/programrequirements/cprresidency_2023.pdf. Accessed October 2, 2023.

[7] Moriates C Dohan D Spetz J et al . Defining competencies for education in health care value: recommendations from the University of California, San Francisco Center for Healthcare Value Training Initiative. Acad Med. 2015;90(4):421–4. 25354077 10.1097/ACM.0000000000000545

[8] Kohlwes RJ Chou CL . A curriculum in medical economics for residents. Acad Med. 2002;77(5):465–6. 12010725 10.1097/00001888-200205000-00040

[9] Dine CJ Miller J Fuld A et al . Educating physicians-in-training about resource utilization and their own outcomes of care in the inpatient setting. J Grad Med Educ. 2010;2(2):175–80. 21975616 10.4300/JGME-D-10-00021.1 PMC2941376

[10] Dewan M Herrmann LE Tchou MJ et al . Development and evaluation of high-value pediatrics: a high-value care pediatric resident curriculum. Hosp Pediatr. 2018;8(12):785–92. 30425056 10.1542/hpeds.2018-0115

[11] Smith CD . Teaching high-value, cost-conscious care to residents: the alliance for Academic Internal Medicine–American College of Physicians Curriculum. Ann Intern Med. 2012;157(4):284–6. 22777503 10.7326/0003-4819-157-4-201208210-00496

[12] Moriates C Soni K Lai A et al . The value in the evidence: teaching residents to “choose wisely.” JAMA Intern Med. 2013;173(4):308–10. 23358796 10.1001/jamainternmed.2013.2286

[13] Hom J Kumar A Evans KH et al . A high value care curriculum for interns: a description of curricular design, implementation and housestaff feedback. Postgrad Med J. 2017;93(1106):725–9. 28663352 10.1136/postgradmedj-2016-134617

[14] Varkey P Murad MH Braun C et al . A review of cost-effectiveness, cost-containment and economics curricula in graduate medical education: teaching cost-effectiveness. J Eval Clin Pract. 2010;16(6):1055–62. 20630001 10.1111/j.1365-2753.2009.01249.x

[15] Schlicher N Haddock A Solnick R et al . EMRA Emergency Medicine Advocacy Handbook. 5th ed. Emergency Medicine Residents’ Association; 2019.

[16] Thomas PA Kern DE Hughes MT et al . Curriculum Development for Medical Education: A Six-Step Approach, 3^rd^ ed. Baltimore, MD: Johns Hopkins University Press; 2016.

[17] Patton MQ .Qualitative research. In: Encyclopedia of Statistics in Behavioral Science; Hoboken, NJ; John Wiley & Sons, 2005.

[18] Lincoln YS Guba EG . Naturalistic Inquiry. Thousand Oaks, CA; Sage Publications, Inc. 1985.

[19] Tong A Sainsbury P Craig J . Consolidated criteria for reporting qualitative research (COREQ): a 32-item checklist for interviews and focus groups. Int J Qual Health Care. 2007;19(6):349–57. 17872937 10.1093/intqhc/mzm042

[20] Stammen LA Stalmeijer RE Paternotte E et al . Training physicians to provide high-value, cost-conscious care: a systematic review. JAMA 2015;314(22):2384–400. 26647260 10.1001/jama.2015.16353

[21] Greysen SR Wassermann T Payne P et al . Teaching health policy to residents—three-year experience with a multi-specialty curriculum. J Gen Intern Med. 2009;24(12):1322–6. 19862580 10.1007/s11606-009-1143-1 PMC2787946

[22] Ryskina KL Smith CD Arora VM et al . Relationship between institutional investment in high-value care (HVC) performance improvement and internal medicine residents’ perceptions of HVC training: Acad Med. 2018;93(10):1517–23. 29697425 10.1097/ACM.0000000000002257 PMC6442932

