# Supplementary material for: Emergency Medicine Resident Needs Assessment and Preferences for a High-value Care Curriculum
Source: West J Emerg Med. 2023 Dec 8;25(1):43–50. doi: 10.5811/westjem.59622 (PMC10777185; doi:10.5811/westjem.59622)
Supplement: Supplementary file 1 [file wjem-25-43-s001.docx]

**APPENDIX**

**Semi structured focus group guide**

**1) Statement of confidentiality: IRB-approved statement read prior to beginning of focus group.**

**2) Introduction of leaders (names, year of training, and primary areas of interest) and participants (names and year of training)**

**3) Topic: HVCHE Knowledge**

- Have you heard the phrase “high value care” used previously? What does this phrase mean to you?
- What do you know about high value cost and basics of health economics.
  - How do you know?
- What do you know about emerging/proposed physician-reimbursement models that prioritize value of care over volume?

**4) Topic: HVCHE Experiences**

- Tell me about time where you had a specific patient- or provider-related experiences where you, the patient, or other providers had concerns related to **high value care**.
  - Describe your interaction with the patient?
  - Describe your interaction with other ED staff?
  - Describe your own feelings about the situation?
  - Could you identify ways ways this situation could have been improved?
- Are there experiences where you have been worried about excess cost (health care spending) as a resident?
  - Based on what you know today, what do you believe may be the cause of the excess costs?
  - Based on what you know today, what are barriers to improvement?

**5) Topic: Educational/Curricular design preferences**

- As a resident, how do you feel about the current education you receive about High Value Care and Health Economics?
- How, as a residency, do you think we could improve our education related to High Value Care and Health Economics?
- Are there specific topics you would like to learn more about and that you believe are important to know as an emergency physician?
- Are you more interested in learning more about current health economics structures or proposed/emerging structures?
  - Follow-up: As a specific example, would you rather learn more about how Emergency Physicians are currently reimbursed, or learn about a new model proposed by American College of Emergency Physicians undergoing regulatory review by the federal government?
- Do you feel comfortable in critically reading an article on health economics and policy?
  - Do you have an interest in improving this?
  - If not, what are ways we can improve this?

**6) Topic: HVCHE Goals/Interests**

- Do you feel like providing high value care is or will be an important part of being an Emergency Physician?
- Do you think being familiar with basic health economics topics is an essential part of Emergency Medicine physician training?
- On the sheet in front of you are 8 possible curriculum formats. Please rank these from 1 to 8 based on what you would prefer:
  - - online modules (e.g., mobile device-friendly website)
    - online interactive forums/apps (e.g., Slack or similar app)
    - series of regularly-assigned non-research readings (e.g., 1-2 page NEJM editorial or public press articles)
    - series of regularly assigned research readings (e.g., primary research articles on cost-effectiveness)
    - grand rounds case-based small group discussions
    - grand rounds didactic lectures
    - morning-report based discussion
    - optional interest group involvement
  - How to distribute, when to distribute, where to distribute?

**Questionnaire/survey**

**1. What is your current year in the emergency department?**

(0) PGY-1
(1) PGY-2
(2) PGY-3
(3) PGY-4

**2. How much previous exposure have you had to the topics of high value care and health economics?**

*(Select all that apply)*

(0) None
(1) Individual study, self-directed learning

(2) Related formal coursework in undergraduate (college) education
(3) Informal education during medical school
(4) Formal coursework (didactics and/or curriculum) in medical school

(5) Degree in related field (Bachelor’s or Master’s in business, health policy, public health, etc.)

Please list amount of exposure (in approx. hours): ______________________________

**3. I feel confident that I can define what high value care means.**

(0) Strongly disagree
(1) Somewhat disagree
(2) Neutral
(3) Somewhat agree

(4) Strongly agree

**4. I feel confident that I know the cost of the care that I provide to patients in the emergency department.**

(0) Strongly disagree
(1) Somewhat disagree
(2) Neutral
(3) Somewhat agree

(4) Strongly agree

**5. I feel confident that I can answer cost-specific and cost-benefit questions that my patients ask during their emergency department visit.**

(0) Strongly disagree
(1) Somewhat disagree
(2) Neutral
(3) Somewhat agree

(4) Strongly agree

**6. I feel confident that I know how insurance companies reimburse for an ED visit.**

(0) Strongly disagree
(1) Somewhat disagree
(2) Neutral
(3) Somewhat agree

(4) Strongly agree

**7. I feel confident that I know how different physician reimbursement models function in Emergency Medicine.**

(0) Strongly disagree
(1) Somewhat disagree
(2) Neutral
(3) Somewhat agree

(4) Strongly agree

**8. It is part of my job as an Emergency Physician to know what high value care means and to be familiar with health economics concepts that affect the cost of care.**

(0) Strongly disagree
(1) Somewhat disagree
(2) Neutral
(3) Somewhat agree

(4) Strongly agree

**9. It is important to have a health economics curriculum in an emergency medicine residency.**

(0) Strongly disagree
(1) Somewhat disagree
(2) Neutral

(3) Somewhat agree

(4) Strongly agree
